# Poly-D,L-Lactic Acid Filler Attenuates Ultraviolet B-Induced Skin Pigmentation by Reducing Destruction of the Basement Membrane

**DOI:** 10.3390/ijms252111568

**Published:** 2024-10-28

**Authors:** Kyung-A Byun, Suk Bae Seo, Seyeon Oh, Jong-Won Jang, Kuk Hui Son, Kyunghee Byun

**Affiliations:** 1 Department of Anatomy & Cell Biology, College of Medicine, Gachon University, Incheon 21936, Republic of Korea; 2 LIBON Inc., Incheon 22006, Republic of Korea; 3 Functional Cellular Networks Laboratory, Lee Gil Ya Cancer and Diabetes Institute, Gachon University, Incheon 21999, Republic of Korea; 4 SeoAh Song Dermatologic Clinic, Seoul 05557, Republic of Korea; 5 Department of Health Sciences and Technology, Gachon Advanced Institute for Health & Sciences and Technology (GAIHST), Gachon University, Incheon 21999, Republic of Korea; 6 Department of Thoracic and Cardiovascular Surgery, Gachon University Gil Medical Center, Gachon University, Incheon 21565, Republic of Korea

**Keywords:** basement membrane destruction, pigmentation, poly-D,L-lactic acid filler

## Abstract

Poly-D,L-lactic acid (PDLLA) filler, which increases volume and collagen synthesis, is used for skin rejuvenation. PDLLA filler also increases M2 macrophages and IL-10. Ultraviolet (UV) radiation induces dermal hyperpigmentation by disrupting the basement membrane (BM), allowing melanin to move into the dermis. Therefore, using UV-irradiated macrophages and animal skin, we determined whether PDLLA filler decreased M1 macrophages and skin inflammation, thereby reducing BM destruction and dermal hyperpigmentation. UV radiation increased the M1 macrophage marker CD86 and TNF-α expression, which was inhibited by the treatment of macrophages with PDLLA. In fibroblasts treated with conditioned medium from UV-irradiated macrophages, NF-κB activity, NLRP3 inflammasome components (NLRP3, ASC, and pro-caspase-1), IL-18, MMP2, and MMP9 increased, but all decreased after PDLLA treatment. Similar to the in vitro study, UV-irradiated mouse skin showed increased CD86, NLRP3, ASC, pro-caspase-1, MMP2, and MMP9, which decreased after PDLLA injection. Disruption of the lamina densa of the BM and dermal pigmentation increased after UV irradiation and decreased after PDLLA injection. In conclusion, PDLLA reduced dermal pigmentation by decreasing BM destruction in UV-irradiated skin. PDLLA has the potential to reduce dermal pigmentation by regenerating the BM.

## 1. Introduction

Ultraviolet (UV) radiation promotes skin inflammation, which is associated with increased proinflammatory cytokines, vasoactive substances, and oxidative stress, as well as increased infiltration of immune cells, such as neutrophils and macrophages [1,2,3,4,5]. UV radiation penetrates the epidermis and the upper dermis, and excessive UV radiation injures keratinocytes, fibroblasts, and melanocytes in the skin [6,7]. UV radiation also increases proinflammatory cytokines such as tumor necrosis factor (TNF)-α and interleukin (IL)-1 in keratinocytes, immune cells, and fibroblasts [8], and TNF-α activates NF-κB [9,10], which increases matrix metalloproteinases (MMPs) [11,12,13,14].

MMPs degrade extracellular matrix (ECM) proteins such as collagen and elastin fibers, resulting in skin wrinkling and photoaging [15,16,17]. MMPs also destroy the basement membrane (BM) [18], a specialized ECM with a thin sheet-like structure [19,20]. The BM layers include the lamina lucida, the lamina densa, and the anchoring fibrils, which consist of several proteins, including collagen IV, collagen VII, laminin-511, laminin-332, nidogen, and perlecan [21].

Keratinocytes comprise 90% of the skin’s protective layer [22]. Melanocytes, located in the basal layer of the epidermis, attach to the BM via collagen IV and laminin [23,24] and protect the skin by absorbing UV light [22]. UV radiation upregulates melanogenesis signaling pathways such as melanocortin-1 receptor (MC1R) signaling and master-regulator microphthalmia-associated transcription factor (MITF) [25], and the melanin is transferred to keratinocytes for UV photoprotection [26]. However, melanocytes that move into the dermis through damaged BM sites cause skin problems, such as melasma [27,28]. Hyperpigmentation is often considered a cosmetic blemish; thus, there are a variety of treatments to reduce melanogenesis [29]. As melanin transfer into the dermis induces persistent and recurring melasma, restoring the BM can reduce the recurrence of hyperpigmentation [30].

Immune cells such as macrophages are involved in photoaging [31]. M1 macrophages produce proinflammatory cytokines such as TNF-α and IL-1, whereas M2 macrophages produce anti-inflammatory cytokines such as IL-10 [32,33,34,35,36]. M1 macrophages increase with aging, especially in sun-exposed skin [31], and lead to an increase in MMPs and a decrease in collagen synthesis in the dermal fibroblast [31].

UV radiation also increases the formation of the nucleotide-binding domain and leucine-rich repeat pyrin-containing protein 3 (NLRP3) inflammasome [37,38,39]. NF-κB promotes translation of NLRP3, thereby increasing the assembly of the inflammasome complex comprising NLRP3, adaptor protein apoptosis-associated speck-like protein containing a C-terminal caspase recruitment domain (ASC), and pro-caspase-1 [40,41,42]. Pro-caspase-1 becomes the active form, caspase-1, via inflammasome formation [43]. Caspase-1 cleaves pro-IL-1β and pro-IL-18 to their active forms, IL-1β and IL-18 [43,44,45]. Caspase-1 also cleaves gasdermin D (GSDMD), generating the N-terminal domain of the protein (GSDMD-NT), which forms pores in the cell membrane, causing pyroptosis [46]. Through these pores, active IL-1β and IL-18 are released into the extracellular space, promoting inflammation [46].

Injectable dermal fillers, which increase the volume and synthesis of ECM materials, are used to rejuvenate skin [47,48,49,50]. Hyaluronic acid, which is one of the natural components of dermis, is highly biocompatible; however, it is degraded quickly [51]. To overcome limitations of short tissue residue time, synthetic biodegradable materials such as poly(lactic acid) (PLA) have been developed as dermal filler [52]. PLA has four types of chiral molecules: poly-D-lactic acid (PDLA), poly-L-lactic acid (PLLA), poly-D,L-lactic acid (PDLLA), and mesoPLA [53,54]. PDLLA is expected to have a superior mechanical stability compared to other chiral molecules due to its stereoisomeric composition and amorphous polymer structure [55].

PDLLA filler increases collagen fiber synthesis [56,57]. We showed previously that PDLLA increases M2 macrophage polarization and IL-10, thereby inducing collagen synthesis in H_2_O_2_-induced senescent fibroblasts and aged mouse skin [58,59]. Because PDLLA increases M2 macrophage polarization in aged skin, we hypothesized that it would decrease M1 macrophages and the release of TNF-α after UV irradiation. We further hypothesized that the decreased release of TNF-α would reduce NF-κB and NLRP3 inflammasome formation, reducing MMPs and BM destruction. The injection of PDLLA into UV-irradiated skin could decrease melanin movement into the dermis via the damaged BM, thereby reducing skin hyperpigmentation. To evaluate our hypotheses, we tested the effects of PDLLA in UV-irradiated mouse skin and in an in vitro model of UV-irradiated macrophages and fibroblasts.

## 2. Results

### 2.1. PDLLA Decreased Expression of CD86 and TNF-α in UV-Irradiated Macrophages

We treated macrophages with UV radiation and found that the expression of TNF-α increased from 10 to 30 s of UV exposure; however, there was no significant difference for exposures of 30 or 40 s (Supplementary Figure S1). Thus, cells were exposed to UV radiation for 20 s in subsequent experiments.

Although UV reaches the upper dermis, it mainly affects keratinocytes in the epidermis [60], suggesting that macrophages could be directly and/or indirectly affected by UV radiation. An indirect effect of UV radiation on macrophages might be caused by keratinocytes. Thus, we compared the expression of M1 macrophage marker CD86 and TNF-α in macrophages irradiated directly with UV radiation vs. indirectly using CM from UV-irradiated keratinocytes (Supplementary Figure S2). We found no significant difference in the expression of CD86 and TNF-α in UV-irradiated macrophage vs. CM-treated macrophage (Supplementary Figure S2B–D). Thus, for simplicity, we exposed macrophages directly to UV radiation in subsequent experiments (Supplementary Figure S3).

**Figure 1:**
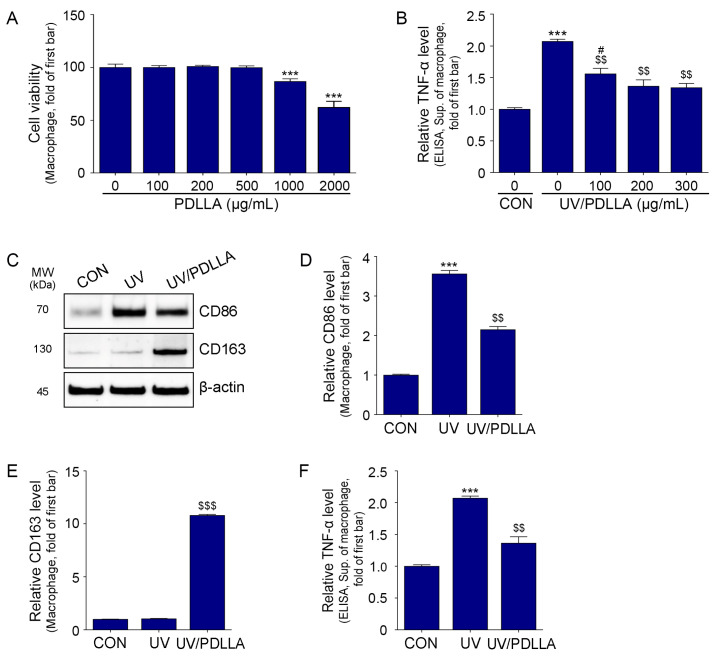
Regulation of CD86, CD163, and TNF-α expression by PDLLA in UV-irradiated macrophages. (**A**) The cell viability by PDLLA in macrophages was measured. (**B**) The expression of TNF-α in the supernatant after treatment of UV-irradiated macrophages with various concentrations of PDLLA was measured by ELISA. (**C**–**E**) The expression of CD86 and CD163 after treatment of UV-irradiated macrophages with 200 μg/mL PDLLA was measured by Western blot. (**F**) The expression of TNF-α in the supernatant after treatment of UV-irradiated macrophages with 200 μg/mL PDLLA was measured by ELISA. Data are presented as the mean ± SD of three independent experiments. ***, *p* < 0.001, vs. first bar; $$, *p* < 0.01, and $$$, *p* < 0.001, vs. second bar; #, *p* < 0.05, vs. fourth bar (Mann–Whitney U test). CD86, cluster of differentiation 86; CD163, cluster of differentiation 163; CON, control; ELISA, enzyme-linked immunosorbent assay; MW, molecular weight; PDLLA, poly-D,L-lactic acid; SD, standard deviation; Sup, supernatant; TNF-α, tumor necrosis factor-α; UV, ultraviolet.

We used cell viability assays to determine the cytotoxicity of PDLLA in macrophages. We found no cytotoxic effects at PDLLA concentrations of up to 500 μg/mL (Figure 1A and Supplementary Figure S3A). Therefore, we measured the effect of PDLLA on melanogenesis in UV-irradiated macrophages. We found that the expression of TNF-α increased after UV irradiation and decreased after treatment with PDLLA at 100, 200, or 300 μg/mL, with similar effects for 200 or 300 μg/mL (Figure 1B). Therefore, we used PDLLA at 200 μg/mL for subsequent experiments.

The expression of CD86 increased after UV irradiation and decreased after treatment with PDLLA. However, the expression of CD163 (an M2 macrophage marker) did not increase significantly after UVB irradiation but did increase after PDLLA treatment (Figure 1C–E). The level of TNF-α in macrophage culture supernatant increased after UV irradiation and decreased after PDLLA treatment (Figure 1F).

### 2.2. PDLLA Decreased the Translocation of NF-κB, Formation of NLRP3 Inflammasome, and Expression of MMP2 and MMP9 in Fibroblasts

**Figure 2:**
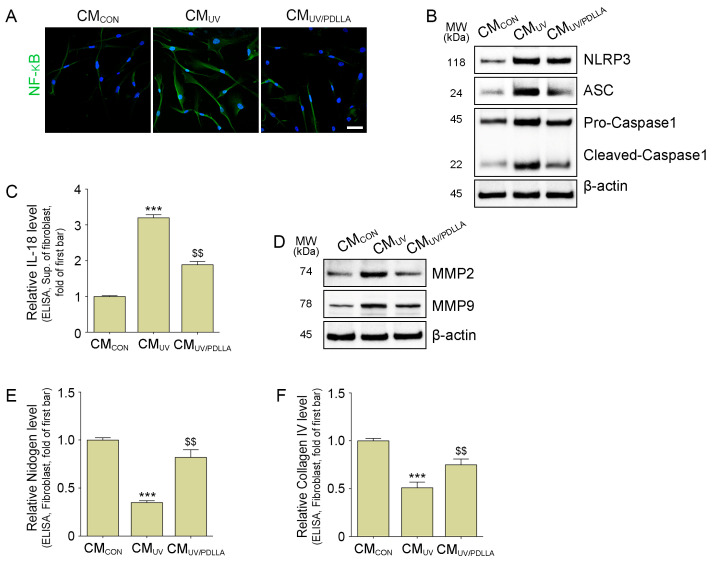
Regulation of the NLRP3 inflammasome and expression of MMPs in fibroblasts treated with CM from UV-irradiated macrophages treated with PDLLA. (**A**) The translocation of NF-κB in fibroblasts treated with CM_CON_, CM_UV_, or CM_UV/PDLLA_ was measured by immunocytochemistry. Scale bar, 50 µm. (**B**) The expression of NLRP3 inflammasome (NLRP3, ASC, and caspase 1) in fibroblasts treated with CM_CON_, CM_UV_, or CM_UV/PDLLA_ was measured by Western blot. (**C**) The expression of IL-18 in the supernatant of fibroblasts treated with CM_CON_, CM_UV_, or CM_UV/PDLLA_ was measured by ELISA. (**D**) The expression of MMPs in fibroblasts treated with CM_CON_, CM_UV_, or CM_UV/PDLLA_ was measured by Western blot. (**E**,**F**) The expression of nidogen and collagen IV fibroblasts treated with CM_CON_, CM_UV_, or CM_UV/PDLLA_ was measured by ELISA. Data are presented as the mean ± SD of three independent experiments. ***, *p* < 0.001, first bar vs. second bar; $$, *p* < 0.01, vs. second bar (Mann–Whitney U test). ASC, apoptosis-associated speck-like protein containing a C-terminal caspase recruitment domain; CM, conditioned medium; CON, control; ELISA, enzyme-linked immunosorbent assay; IL-18, interleukin-18; MMP, matrix metalloproteinase; MW, molecular weight; NF-κB, nuclear factor kappa-light-chain-enhancer of activated B cells; NLRP3, nucleotide-binding domain and leucine-rich repeat pyrin-containing protein 3; PDLLA, poly-D,L-lactic acid; SD, standard deviation; Sup, supernatant; UV, ultraviolet.

We hypothesized that UV-irradiated macrophages had increased secretion of TNF-α, which caused dermal fibroblasts to destroy the BM by increasing MMPs. Thus, we treated fibroblasts with CM from UV-irradiated macrophages (CM_UV_) or CM from PDLLA-treated UV-irradiated macrophages (CM_UV/PDLLA_) (Supplementary Figure S4). CM_UV_ increased the translocation of NF-κB in the fibroblast, but CM_UV/PDLLA_ decreased translocation (Figure 2A and Supplementary Figure S5A). The components of NLRP3 inflammasome (NLRP3, ASC, and pro-caspase 1) increased in fibroblasts with CM_UV_ and decreased with CM_PDLLA_ treatment (Figure 2B and Supplementary Figure S5B–D). Similarly, the expression of cleaved-caspase 1 and IL-18 increased, as did the expression of MMP2 and MMP9, which decreased with CM_UV/PDLLA_ treatment (Figure 2B–D and Supplementary Figure S5E–G). In contrast, the expression of nidogen and collagen IV, which are the main components of the BM, decreased in fibroblasts with CM_UV_ and increased with CM_UV/PDLLA_ treatment (Figure 2E,F).

### 2.3. PDLLA Decreased Expression of CD86 and TNF-α in UV-Irradiated Mouse Skin

**Figure 3:**
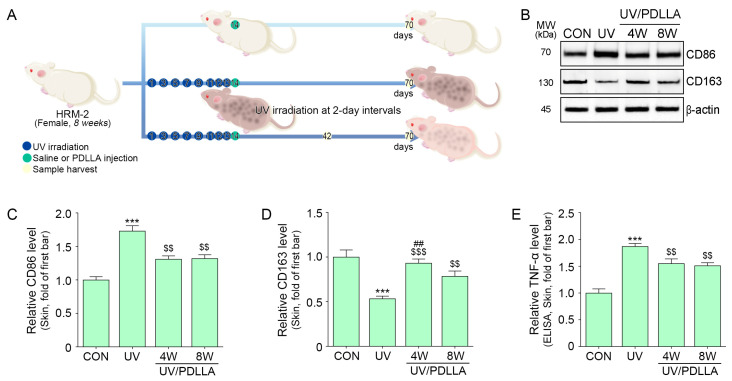
Regulation of CD86, CD163, and TNF-α expression by PDLLA in UV-irradiated mouse skin. (**A**) Schematic diagram of the treatment with PDLLA in UV-irradiated mouse skin. The green circles indicate material injection time, and yellow circles are sampling days. (**B**–**D**) The expression of CD86 and CD163 by PDLLA in UV-irradiated mouse skin was measured by Western blot. (**E**) The expression of TNF-α by PDLLA in UV-irradiated mouse skin was measured by ELISA. Data are presented as the mean ± SD of three independent experiments. ***, *p* < 0.001, first bar vs. second bar; $$, *p* < 0.01, and $$$, *p* < 0.001 vs. second bar; ##, *p* < 0.01, vs. fourth bar (Mann–Whitney U test). CD86, cluster of differentiation 86; CD163, cluster of differentiation 163; CON, control; ELISA, enzyme-linked immunosorbent assay; MW, molecular weight; PDLLA, poly-D,L-lactic acid; SD, standard deviation; TNF-α, tumor necrosis factor-α; UV, ultraviolet W, weeks.

In the in vivo mouse skin model (Figure 3A), the expression of CD86 increased after UV irradiation and decreased after subsequent PDLLA injection (Figure 3B,C). In contrast, the expression of CD163 decreased after UV irradiation and increased after subsequent PDLLA injection. The expression of CD163 was higher at 4 weeks after the PDLLA injection than at 8 weeks after PDLLA injection (Figure 3B,D). The expression of TNF-α increased after UV irradiation and decreased similarly at 4 weeks and 8 weeks after PDLLA injection (Figure 3E).

### 2.4. PDLLA Decreased Translocation of NF-κB, Formation of the NLRP3 Inflammasome, and Expression of MMP2 and MMP9 in UVB-Irradiated Mouse Skin

**Figure 4:**
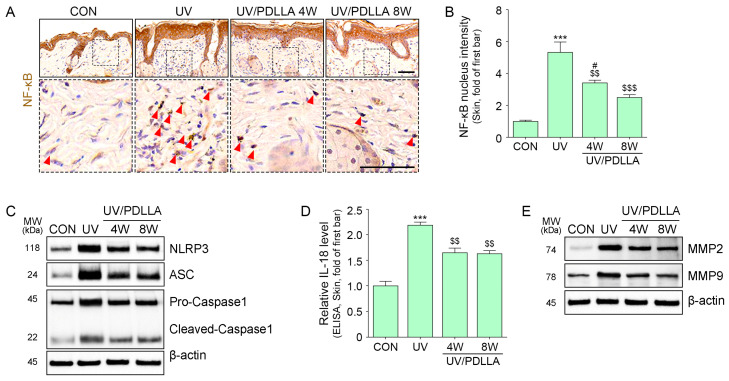
Regulation of NLRP3 inflammasome and MMPs expression by PDLLA in UVB-irradiated mouse skin. (**A**,**B**) The nucleus-positive cells of NF-κB after PDLLA injection in UVB-irradiated mouse skin were measured by immunohistochemistry. The red arrow indicates NF-κB nucleus-positive cell. Scale bar, 60 µm. (**C**) The expressions of the NLRP3 inflammasome, (**D**) IL-18, and (**E**) MMPs after PDLLA injection in UVB-irradiated mouse skin were measured by Western blot or ELISA. Data are presented as the mean ± SD of three independent experiments. ***, *p* < 0.001, first bar vs. second bar; $$, *p* < 0.01, and $$$, *p* < 0.001 vs. second bar; #, *p* < 0.05, vs. fourth bar (Mann–Whitney U test). ASC, apoptosis-associated speck-like protein containing a C-terminal caspase recruitment domain; CON, control; ELISA, enzyme-linked immunosorbent assay; IL-18, interleukin-18; MMP, matrix metalloproteinase; MW, molecular weight; NF-κB, nuclear factor kappa-light-chain-enhancer of activated B cells; NLRP3, nucleotide-binding domain and leucine-rich repeat pyrin-containing protein 3; PDLLA, poly-D,L-lactic acid; SD, standard deviation; UV, ultraviolet; W, weeks.

Expression of NF-κB lesions increased after UVB irradiation and decreased 4 weeks after the injection of PDLLA, with a further decrease after 8 weeks (Figure 4A,B). Similarly, the expression of NLRP3, ASC, pro-caspase 1, cleaved caspase 1, and IL-18 increased after UVB irradiation and decreased 4 weeks after injection of PDLLA, with no further decrease after 8 weeks (Figure 4C,D and Supplementary Figure S6A–D). The expression of MMP2 and MMP9 increased after UVB irradiation and decreased 4 weeks after injection of PDLLA, with a further decrease after 8 weeks (Figure 4E and Supplementary Figure S6E,F).

### 2.5. PDLLA Decreased BM Destruction and Melanin Accumulation in UVB-Irradiated Skin

**Figure 5:**
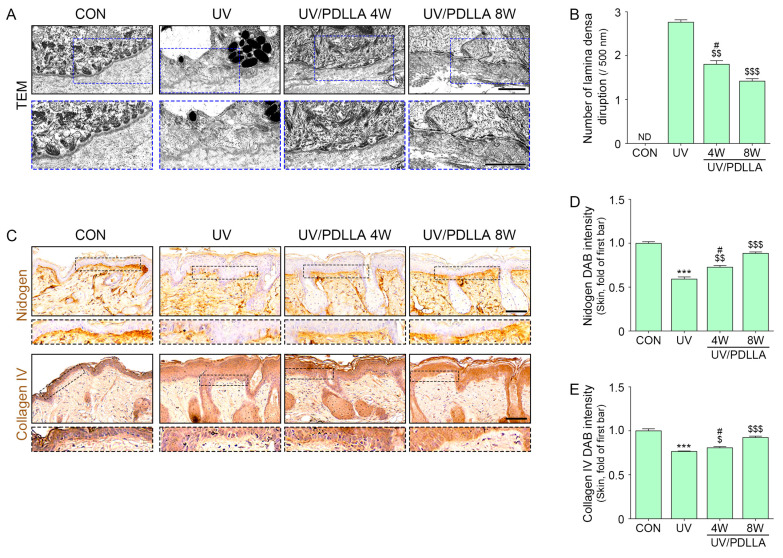
Regulation of BM destruction by injection of PDLLA into UVB-irradiated mouse skin. (**A**) BM destruction was demonstrated by TEM. The red mark indicates lamina densa with disruptions. The blue-dash boxes indicate magnification. Scale bar, 1 µm. (**B**) Quantitative assessment of TEM image presented in (**A**). (**C**) The expression of nidogen and collagen IV after injection of PDLLA in UVB-irradiated mouse skin was measured using immunohistochemistry. Scale bar, 60 µm. (**D**,**E**) Quantitative assessment of immunohistochemistry data presented in (**C**). Data are presented as the mean ± SD of three independent experiments. ***, *p* < 0.001, first bar vs. second bar. $, *p* < 0.05; $$, *p* < 0.01; and $$$, *p* < 0.001, vs. second bar. #, *p* < 0.01, vs. fourth bar (Mann–Whitney U test). CON, control; DAB, 3, 3'-diaminobenzidine; ND, not done or not detectable; PDLLA, poly-D,L-lactic acid; SD, standard deviation; TEM, transmission electron microscopy; UV, ultraviolet; W, weeks.

Disruption of the lamina densa occurs in sun-exposed human or mouse skin [61,62]. TEM images showed that lamina densa disruption lesions increased after UVB irradiation and decreased 4 weeks after injection of PDLLA, with a further decrease after 8 weeks (Figure 5A,B). In contrast, the expression of nidogen and collagen IV decreased after UVB irradiation and increased 4 weeks after the injection of PDLLA, with a further increase after 8 weeks (Figure 5C–E).

**Figure 6:**
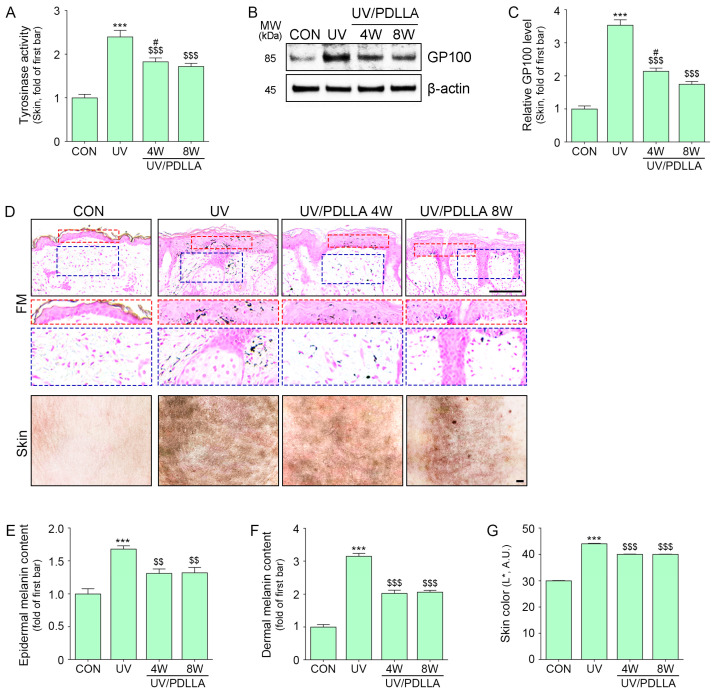
Regulation of melanin accumulation by injection of PDLLA in UVB-irradiated mouse skin. (**A**) Tyrosinase activity by PDLLA in UV-irradiated mouse skin was measured. (**B**,**C**) The expression of GP100 after injection of PDLLA in UVB-irradiated mouse skin was measured using Western blot. Scale bar, 100 µm. (**D**) Melanin content after injection of PDLLA in UVB-irradiated mouse skin was determined by Fontana-Masson stain in the epidermis (red dashes boxes) and dermis (blue dashes boxes) or skin color of mouse skin. Scale bar, 100 µm. (**E**–**G**) Quantitative assessment of Fontana-Masson and skin-color data presented in Figure 6D. ***, *p* < 0.001, first bar vs. second bar; $$, *p* < 0.01, and $$$, *p* < 0.001, vs. second bar; #, *p* < 0.01, vs. fourth bar (Mann–Whitney U test). CON, control; FM, Fontana-Masson; GP100, glycoprotein 100; PDLLA, poly-D,L-lactic acid; SD, standard deviation; UV, ultraviolet; W, weeks.

Tyrosinase activity and expression of glycoprotein 100 (GP100), which both increase during melanogenesis, were evaluated. Both were increased after UV irradiation and decreased after PDLLA injection (Figure 6A–C).

Melanin content in the epidermis and dermis, as measured by Fontana-Masson staining, increased after UV irradiation and decreased after PDLLA injection. Skin color, as measured by colorimeter, increased after UVB irradiation and decreased similarly at 4 and 8 weeks after injection of PDLLA (Figure 6D–G).

## 3. Discussion

Hyperpigmentation is characteristic of post-inflammatory hyperpigmentation (PIH), melasma, and solar lentigines [63,64]. PIH is caused by inflammation in the epidermis and dermis [65]. In the epidermis, increased inflammatory factors lead to melanogenesis in the melanocyte [66]. In the dermis, inflammation injures the basal keratinocytes, releasing melanin [67], which is phagocytosed by macrophages generating melanophages [67]. Melanophages that remain in the upper dermis produce persistent blue-gray patches [67]. When the BM structure functions well, it serves as a barrier between the epidermis and dermis, preventing the movement of melanophages or melanosome-containing fragments from keratinocytes across the BM into the dermis [68,69,70]. The accumulation of dermal melanin under solar lentigines skin results in decreased collagen IV expression and increased MMPs compared with non-solar lentigines skin [64]. As melanin phagocytosis in the dermis is much slower than in the epidermis, dermal melanin in the dermis is eliminated more slowly than in the epidermis [71]. Thus, protection of the BM from UV radiation injury or regeneration of the BM decreases dermal hyperpigmentation [64].

We found previously that PDLLA increases M2 polarization and IL-10 in aged mice mouse skin [59], suggesting that PDLLA decreases M1 polarization, which reduces proinflammatory cytokines such as TNF-α. Here, we show that that UVB radiation increased the expression of CD86 and decreased the expression of CD 163 in macrophages, suggesting that UVB radiation induced macrophage polarization toward M1. UVB radiation also increased TNF-α; however, PDLLA decreased M1 polarization and TNF-α in the UVB-irradiated macrophage.

NLRP3 inflammasomes are associated with inflammatory skin diseases, including vitiligo, atopic dermatitis, and psoriasis [72]. UV radiation increases NLRP3 inflammasome formation in the keratinocyte [39]. Photoaged skin accumulates advanced glycated end products in the dermis that stimulate NLRP3 inflammasome formation in the dermal fibroblast [73]. Moreover, increased NLRP3 inflammasomes stimulate melano-genesis in the dermal fibroblast [73]. Consistent with previous studies, here, we show increased NLRP3 inflammasome formation, as well as increased cleaved caspase-1 and IL-18 in fibroblasts treated with CM from UVB-irradiated macrophages. The addition of CM from PDLLA-treated, UVB-irradiated macrophages reversed these increases in fibroblasts.

NF-κB mediates the inflammatory priming signal, promoting the formation of the NLRP3 inflammasome [74]. TNF-α stimulates NF-κB, which activates MMPs, as well as the NLRP3 inflammasome [75,76]. Here, we show that fibroblasts treated with CM from UVB-irradiated macrophages had increased NF-κB, MMP2, and MMP9 expression, but treatment with CM from PDLLA-treated, UVB-irradiated macrophages decreased that expression. In contrast, nidogen and collagen IV expression showed the opposite effect, and PDLLA increased in the fibroblast.

Consistent with the in vitro studies, we found that UVB-irradiated mouse skin had increased the expression of CD86 and TNF-α, as well as increased the translocation of NF-κB, the expression of NLRP3 inflammasome components, and IL-18. These all decreased after the injection of PDLLA into the UVB-irradiated skin. MMP2 and MMP9 also increased in UVB-irradiated skin; however, nidogen and collagen IV decreased. UV induces BM injuries; thus, controlling MMPs improves the repair of the BM [77]. For example, MMP inhibitors increase the formation of hemidesmosomes and anchoring fibrils of the BM [77]. Purified laminin 332 increases the production of BM components and the formation of hemidesmosome-like structures [78,79]. After PDLLA injection in the UVB-irradiated skin, we found increased nidogen and collagen IV expression, as well as decreased lamina densa disruption, decreased dermal melanin accumulation, and lightening of skin color.

Conventional melasma or PIH treatments include topical whitening agents, such as tyrosinase inhibitors and hydroquinone, mequinol, and retinoids [80,81]. However, these agents may not decrease melanin effectively and can irritate the skin [80,81]. Chemical peels can remove melanin-containing epidermal cells; however, peels irritate the skin and may result in hyperpigmentation [80,81]. Laser therapy may also induce paradoxical hyperpigmentation [82,83].

PDLLA fillers are often used to rejuvenate skin by increasing ECM components such as collagen. However, our study showed that PDLLA decreased UVB-induced skin inflammation and decreased BM destruction, resulting in decreased skin pigmentation, including dermal pigmentation. The PDLLA-induced decrease in MMP2 and MMP9 and the increase in nidogen and collagen IV were measured 8 weeks after injection and will likely be sustained for more than 6 months. PDLLA injected into the nasolabial fold in humans increases collagen for 6 months [84]. In mouse skin, a PDLLA injection lasts for 20 weeks and increases collagen synthesis [56]. Because topical agents often induce skin irritation, they are not ideal long-term treatments. For example, topical hydroquinone treatment is not recommended for longer than 6 months [85]. An effective treatment, including repair of the BM to prevent further dermal melanogenesis, would also prevent the recurrence of dermal pigmentation.

We demonstrated that PDLLA decreased skin inflammation, as well as BM destruction; thus, it could effectively inhibit melanin migration. Based on an in vitro and animal study, we were able to obtain the clue that PDLLA can be used in humans to reduce pigmentation by decreasing BM destruction. However, the possibility of PDLLA as anti-pigmentation in humans should be evaluated in human study.

There are several limitations in this study. First, we did not determine the mechanism by which PDLLA decreased M1 polarization. Second, our mouse studies lasted only 8 weeks. The long-term effects of PDLLA should be evaluated in a future study. However, our study suggests that PDLLA not only increases collagen synthesis but effectively re-duces dermal pigmentation.

## 4. Materials and Methods

### 4.1. PDLLA Preparation

PDLLA (VAIM Co., Ltd., Daejeon, Republic of Korea) was dissolved in a mixture of ethylene carbonate and dimethyl sulfoxide (Sigma-Aldrich, St. Louis, MO, USA) in a 1:9 ratio. This solution was sprayed into cold n-hexane (below −10 °C; Sigma-Aldrich). The solvent/polymer mixture was combined with distilled water (DW) and filtered to remove the solvent, yielding solid PDLLA particles measuring from 10 to 30 µm. These PDLLA particles were dried and mixed with a 0.6% hyaluronic acid solution at a ratio of 17:3. The mixture was aliquoted into 10 mL vials, lyophilized, and sterilized with gaseous ethylene oxide before use [58,59].

### 4.2. In Vitro Experiments

#### 4.2.1. Cell Culture

THP-1 human monocytes were obtained from the American Type Culture Collection (Manassas, VA, USA) and cultured in RPMI-1640 medium (Welgene, Gyeongsan, Republic of Korea) at 37 °C with 5% CO_2_.

HaCaT human keratinocytes were provided and grown by Professor Jeong Hee Hong’s team at Gachon University. These cells were cultured in Dulbecco’s modified Eagle’s medium (HyClone, Logan, UT, USA) at 37 °C with 5% CO_2_.

CCD-986Sk human fibroblasts were acquired from Korean Cell Line Bank (Seoul, Republic of Korea) and cultured in Iscove’s Modified Dulbecco’s Medium (Gibco, Waltham, MA, USA) at 37 °C with 5% CO_2_.

#### 4.2.2. Experimental Design of In Vitro

In vitro experiments included five stages to evaluate the effects of PDLLA on macrophages and fibroblast. (1) To determine the optimal UV dose for PDLLA experiments, a macrophage model was created by treating monocytes with 100 nM phorbol 12-myristate 13-acetate (PMA) and exposed to a UV lamp (G15T8E, Sankyo, Yokohama, Japan) at a height of 40 cm for 10, 20, 30, or 40 s to create a macrophage model (Supplementary Figure S1A). (2) To compare macrophages directly exposed to UV radiation with those treated with the supernatant, i.e., conditioned medium (CM), from UV-irradiated keratinocytes, a second macrophage model was designed. In this model, monocytes were treated with 100 nM PMA and with CM from UV-irradiated keratinocytes. Macrophage cell lysates and CM were collected for protein analysis (Supplementary Figure S2A). (3) To determine the cytotoxicity of PDLLA, monocytes were treated with 100 nM PMA and incubated for 24 h with phosphate-buffered saline (PBS) or 100, 200, 500, 1000, or 2000 μg/mL PDLLA (Supplementary Figure S3A). (4) To determine the optimal concentration of PDLLA, we treated monocytes with 100 nM PMA, exposed them to UV radiation, and incubated them for 48 h with phosphate-buffered saline (PBS) or with 100, 200, or 300 μg/mL of PDLLA. Control cells not exposed to UV radiation were incubated with PBS for 48 h. Following incubation, we collected cell lysates for protein analysis, and supernatants were harvested to be used as CM for fibroblast treatment (Supplementary Figure S3B). (5) A fibroblast model was established by treating fibroblasts for 48 h with CM from three treatment groups of macrophages. Cell lysates from all fibroblast groups were subsequently collected for protein analysis (Supplementary Figure S4).

### 4.3. In Vivo Experiments

#### 4.3.1. Mouse Model and Maintenance

Female HRM-2 mice, 6 weeks old, were obtained from the Central Laboratory Animal Center (Incheon, Republic of Korea) and acclimated in our facility for 2 weeks prior to the experiments. The mice were housed in a controlled environment with a constant temperature of 20–24 °C and humidity levels of 45–55%, with free access to food and water. This study was conducted with the approval of the Gachon University Animal Experiment Ethics Committee (IACUC, approval number LCDI-2023-0155).

#### 4.3.2. Experimental Design of In Vivo

The stabilized mice were randomly divided into four groups. One of these groups was a control group that was not exposed to UV (Group 1). Three of these groups were irradiated with UV light, as we described previously [86]. In brief, a UV lamp (Sankyo) with a peak wavelength of 306 nm was used to apply 200 mJ/cm^2^ of UV radiation to the backs of the mice every other day for 10 days, followed by daily UV irradiation for three consecutive days. Afterward, either normal saline (Group 2) or 10 mg/mL of PDLLA (Group 3, 4) was administered once to a 2 cm × 2 cm on the backs of the mice, using a 27G needle, and UV irradiation continued every other day. The total injection volume for all solutions was 500 μL. After 28 (Group 3) or 42 days (Group 4), the mouse skin was harvested for analysis.

#### 4.3.3. Skin Color

Skin color was assessed using a CR-10 color reader (Konica Minolta Sensing, Inc., Sakai, Osaka, Japan), with L* (brightness) values measured in the CIELAB color space (International Commission on Illumination, Vienna, Austria). The measurements were averaged for 10 readings per mouse taken on the 42nd or 70th day (28 or 42 days after the start of treatment) following the initial UV radiation exposure.

### 4.4. Sample Preparation

#### 4.4.1. Protein Isolation

Protein extraction was performed using the EzRIPA buffer kit (ATTO Corporation, Tokyo, Japan), following the manufacturer’s instructions. Cells were washed with PBS and scraped into 0.4 mL of RIPA buffer. For skin samples, 50 mg of tissue was cut into small pieces, diluted with 0.6 mL of RIPA buffer, and homogenized 10 cycles (40 s homogenization/60 s rest). The samples were then incubated on ice for 10 min for protein solubilization. Cell and tissue samples were further sonicated at high power (10 s sonication/60 s rest) and centrifuged at 14,000× *g* for 15 min at 4 °C. Protein concentration was determined using a bicinchoninic acid assay kit (Thermo Fisher Scientific, Waltham, MA, USA).

#### 4.4.2. Paraffin-Embedded Block

Skin tissue was fixed in cold 4% paraformaldehyde (Sigma-Aldrich) for 72 h, placed in a cassette, and washed with DW. The sample was processed in a tissue processor (Leica, Wetzlar, Germany), where it was dehydrated in 95% and 99% ethanol (Duksan, Ansan, Republic of Korea), followed by xylene (Duksan), and then infiltrated with paraffin (Leica). The paraffin-soaked tissue was embedded into paraffin blocks, using an embedding machine. The blocks were sectioned into 7 µm thick slices, using a microtome (Leica), and attached to coated slides by incubating overnight at 60 °C.

### 4.5. Cell Viability

To assess the cytotoxicity of PDLLA, we seeded monocytes into a 96-well plate at a density of 1 × 10^4^ cells per well and treated with 100 nM PMA. Once the wells were fully confluent, the cells were exposed to PDLLA at concentrations of 100, 200, 500, 1000, and 2000 μg/mL for 24 h. After treatment, the medium was removed, and the cells were washed with DPBS (Gibco). Next, 10 µL of CCK-8 reagent (TransGen Biotech Co., Ltd., Beijing, China) and 90 µL of growth medium were added to each well, and the cells were incubated at 37 °C for 2 h. Optical density at 450 nm was measured using a microplate reader. Each concentration was tested in triplicate.

### 4.6. Enzyme-Linked Immunosorbent Assay (ELISA)

Microplates were incubated overnight at 4 °C with 100 nM carbonate–bicarbonate buffer (pH 9.6) and then washed three times with 0.1% Tween 20 in PBS (TPBS) to remove any unbound material. To block non-specific protein binding, we incubated the microplates with 5% skim milk (LPS Solution) in 0.1% TPBS overnight at 4 °C. Following three washes with 0.1% TPBS, 100 μg of the protein sample or 100 μL of CM was added to each well and incubated overnight at 4 °C. After washing with 0.1% TPBS, the plates were incubated overnight at 4 °C with the primary antibody diluted in PBS (Supplementary Table S1). After washing with 0.1% TPBS, a horseradish peroxidase-conjugated secondary antibody (1:10,000; Vector Laboratories, Newark, CA, USA) was added and incubated at room temperature for 3 h. To detect protein expression, tetramethylbenzidine (TMB) solution (Sigma-Aldrich) was added to each well and incubated for 10 min at room temperature. The reaction was stopped with 1 M sulfuric acid (Sigma-Aldrich). Finally, optical density at 450 nm was measured using a microplate reader.

### 4.7. Western Blot

Fifty micrograms of protein from cell lysates or skin was denatured with 4× LDS sample buffer (Thermo Fisher Scientific) and 10× sample-reducing agent (Thermo Fisher Scientific) at 70 °C for 10 min. The denatured sample was separated protein in 10% sodium dodecyl sulfate–polyacrylamide gel electrophoresis at 200 V, using MOPS buffer (Invitrogen, Waltham, MA, USA). The separated proteins were transferred to a polyvinylidene fluoride membrane (Millipore, Burlington, MA, USA), using a semi-dry transfer system, at 1 A for 10 min. After the membrane blocked with 5% skim milk (LPS Solution, Daejeon, Republic of Korea) in 0.1% Tween 20 (SPL, Pocheon, Republic of Korea) in Tris-buffered saline (TTBS) at room temperature for 1–3 h. After three washes with 0.1% TTBS, the membrane was incubated with the appropriately diluted primary antibody overnight at 4 °C (Supplementary Table S1). Following another three washes with 0.1% TTBS, the membrane was incubated with a horseradish peroxidase-conjugated secondary antibody (1:10,000; Vector Laboratories, Newark, CA, USA) for 1 h at room temperature. Protein bands were visualized using a ChemiDoc Imaging System (Bio-Rad, Hercules, CA, USA).

### 4.8. Staining

#### 4.8.1. Immunocytochemistry (ICC)

The seeded cells were fixed in 4% paraformaldehyde (Sigma-Aldrich) for 15 min at room temperature in 8-well Lab-Tek II chamber slides (Nunc™, Sigma-Aldrich). After three PBS washes, the cells were blocked with serum solution for 1 h at room temperature, and the cells were incubated with the primary antibody overnight at 4 °C (Supplementary Table S1). After washing with PBS, the cells were incubated with the Alexa Fluor™ 488 secondary antibodies (Invitrogen) for 1 h at room temperature. For counterstaining, the cells were incubated with 1 µg/mL of 4′,6-diamidino-2-phenylindole (Sigma-Aldrich) for 30 s and mounted using a Vectashield mounting solution (Vector Laboratories). Finally, analysis was done by using confocal microscope (LSM-710; Carl Zeiss, Jena, Germany) at Core-facility for Cell to In-vivo imaging.

#### 4.8.2. Immunohistochemistry (IHC)

The deparaffinized slides were blocked with serum solution for 1 h at room temperature, and the slides were incubated with the primary antibody overnight at 4 °C (Supplementary Table S1). After washing with PBS, the slides were incubated with the biotinylated secondary antibody (Vector Laboratories) for 1 h at room temperature. The slides were then rinsed with PBS, incubated with an ABC reagent (Vector Laboratories), washed, and incubated with a 3,3′-diaminobenzidine solution (Sigma-Aldrich) for 5 min, resulting in a brown reaction. For counterstaining, the slides were incubated with hematoxylin (KPNT, Cheongju, Republic of Korea) for 30 s, washed with DW, dehydrated, and mounted using a DPX mounting solution (Sigma-Aldrich). Finally, the stained tissue was scanned using a slide scanner (Motic Scan Infinity 100; Motic, Beijing, China), and images were captured.

#### 4.8.3. Fontana-Masson Staining

Fontana-Masson staining was carried out following the manufacturer’s protocol (Scytek, Logan, UT, USA). Briefly, the deparaffinized slides were then incubated in Fontana ammoniacal silver solution for 30 min at 60 °C. After rinsing three times with DW, we removed non-melanin-stained areas using 0.2% gold chloride solution and 5% sodium thiosulfate solution. The nuclei were stained with Nuclear Fast Red solution, and the sections were dehydrated and mounted with DPX mounting solution (Sigma-Aldrich). The stained tissue was scanned with a slide scanner (Motic Scan Infinity 100), and images were captured.

### 4.9. Transmission Electron Microscopy (TEM) Imaging

The tissue was cut into 1 mm × 1 mm pieces and fixed in 2% glutaraldehyde/2% paraformaldehyde in a 0.1 M phosphate buffer (pH 7.4) for 24 h. After washing with the same buffer, the skin sections were fixed in 1% OsO_4_ in a 0.1 M phosphate buffer for 2 h and then dehydrated through a graded ethanol series (50, 60, 70, 80, 90, 95, and 100%; 10 min each). The sections were subsequently permeated with propylene oxide for 10 min, embedded using the Poly/Bed 812 kit (Polysciences, Inc., Warrington, PA, USA) for 12 h, and polymerized in an electron microscope oven at 65 °C for 12 h. The resulting block was sectioned into 200 nm slices, using a diamond knife, on an ultramicrotome and stained with toluidine blue for light microscopy. These sections were further thinned to 80 nm, placed on a copper grid, stained with 3% uranyl acetate for 30 min, and double-stained with 3% lead citrate for 7 min [87].

### 4.10. Tyrosinase Activity

Tyrosinase activity was carried out following the manufacturer’s protocol (Abcam, Waltham, MA, USA). Briefly, the skin samples were mixed substrate into a 96-well plate. The plate was incubated at 37 °C for 2 h. Optical density at 510 nm was measured using a microplate reader. Each concentration was tested in triplicate.

### 4.11. Quantitative and Statistical Analysis

For quantitative analysis, intensity was measured using ImageJ software version 1.53s (NIH). Each group was compared with the control sample [88,89]. The Kruskal–Wallis test was used to compare the groups, with post hoc analyses conducted using the Mann–Whitney U test. Results are presented as mean ± standard deviation (SD). Statistical analyses were carried out using SPSS version 26 (IBM, Armonk, NY, USA). Statistical significance is noted in each figure legend.

## 5. Conclusions

PDLLA decreased M1 polarization and TNF-α, which was also associated with changes in NF-κB activity and the NLRP3 inflammasome in UVB-irradiated skin. PDLLA also decreased MMP2 and MMP9 and increased the expression of the BM components nidogen and collagen IV. Those changes were accompanied by decreased dermal pigmentation and a lightening of skin color.

## Data Availability

All data are contained within this article.

## Supplementary Materials

The following supporting information can be downloaded at https://www.mdpi.com/article/10.3390/ijms252111568/s1.
