# Supplementary material for: Poly-D,L-Lactic Acid Filler Attenuates Ultraviolet B-Induced Skin Pigmentation by Reducing Destruction of the Basement Membrane
Source: Int J Mol Sci. 2024 Oct 28;25(21):11568. doi: 10.3390/ijms252111568 (PMC11546917; doi:10.3390/ijms252111568)
Supplement: Supplementary file 1 [file ijms-25-11568-s001.zip › ijms-3251828-SI.pdf]

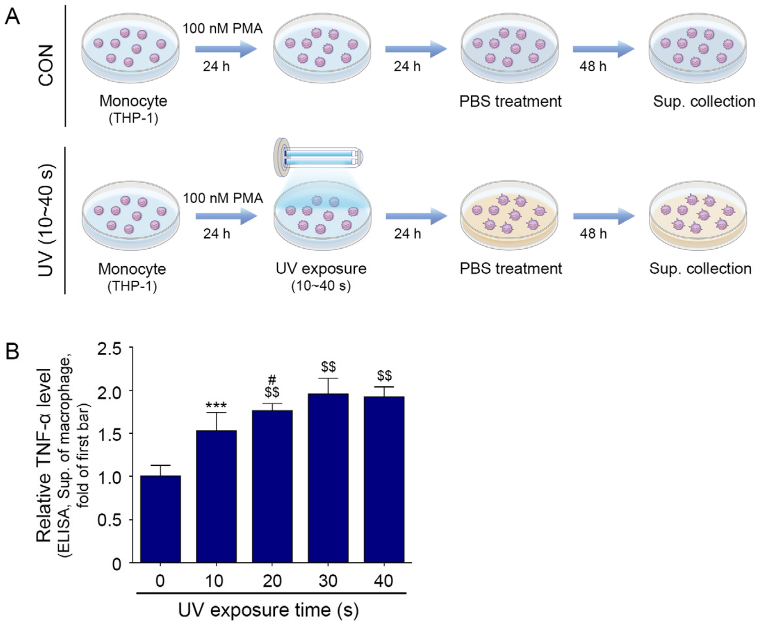


**Figure S1.** Regulation of TNF-$\alpha$ expression in UV-irradiated macrophages. (**A**) Schematic diagram of macrophage induction of THP-1 cells with PMA and treatment of UV-irradiated macrophages. (**B**) TNF-$\alpha$ in the supernatant after treatment of UV-irradiated macrophages was measured using an ELISA. Data are presented as the mean ± SD of three independent experiments. ***, $p < 0.001$, vs. first bar; $$, $p < 0.01$, vs. second bar; #, $p < 0.05$, vs. fourth bar (Mann–Whitney U test). ELISA, enzyme-linked immunosorbent assay; PBS, phosphate-buffered saline; PMA, phorbol 12-myristate 13-acetate; SD, standard deviation; Sup, supernatant; TNF-$\alpha$, tumor necrosis factor-$\alpha$; UV, ultraviolet.

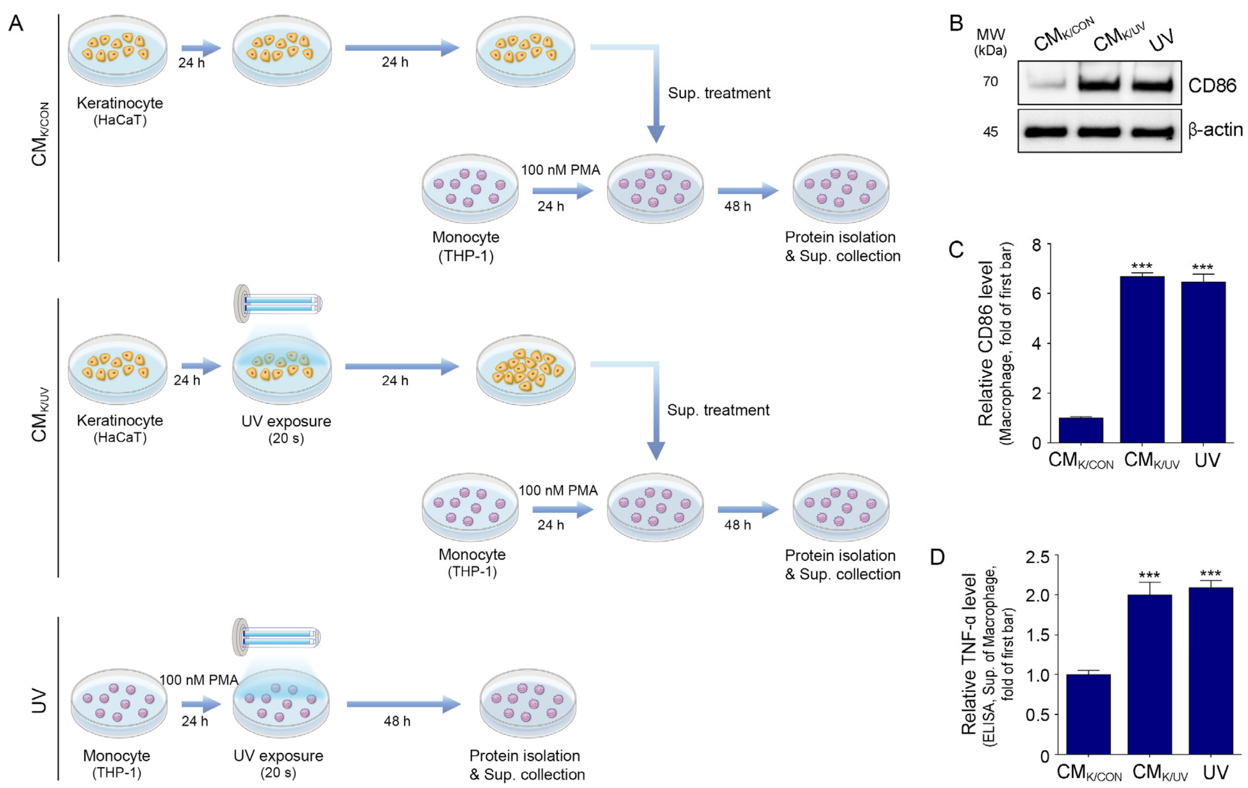


**Figure S2.** Comparison of CD86 and TNF-$\alpha$ expression in macrophages irradiated directly with UVB vs. macrophages treated with CM. (**A**) Schematic diagram of the treatment of UV-irradiated or CM-treated macrophages. (**B**,**C**) The expression of CD86 in UV-irradiated or CM-treated macrophages was measured by western blot. (**D**) The expression of TNF-$\alpha$ in the supernatant after treatment of UV-irradiated or CM-treated macrophages was measured by ELISA. Data are presented as the mean ± SD of three independent experiments. ***, $p < 0.001$, vs. first bar (Mann–Whitney U test). CD86, cluster of differentiation 86; CM, conditioned medium; ELISA, enzyme-linked immunosorbent assay; MW, molecular weight; PBS, phosphate-buffered saline; PMA, phorbol 12-myristate 13-acetate; SD, standard deviation; Sup, supernatant; TNF-$\alpha$, tumor necrosis factor-$\alpha$; UV, ultraviolet.

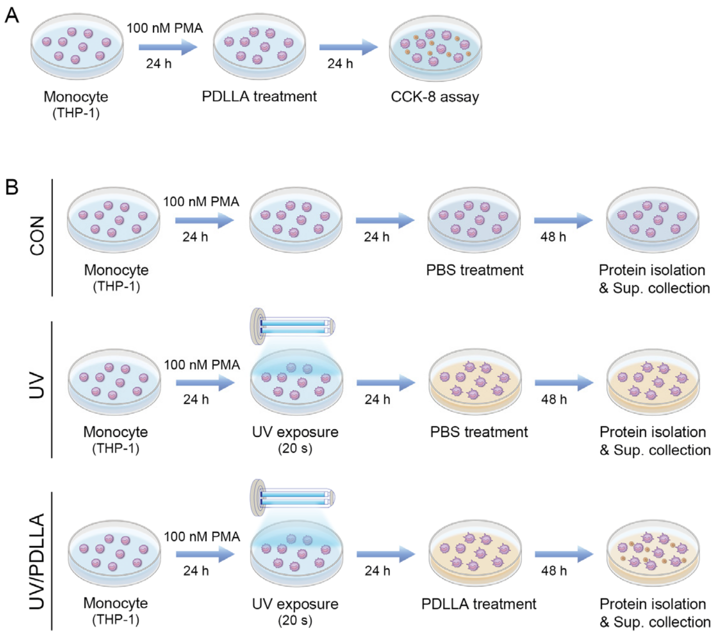


**Figure S3.** Schematic diagram of PDLLA treatment of macrophages or UV-irradiated macrophages. (**A**) Schematic diagram of PDLLA treatment of macrophages. (**B**) Schematic diagram of PDLLA treatment of UV-irradiated macrophages. PBS, phosphate-buffered saline; PMA, phorbol 12-myristate 13-acetate; Sup, supernatant; UV, ultraviolet.

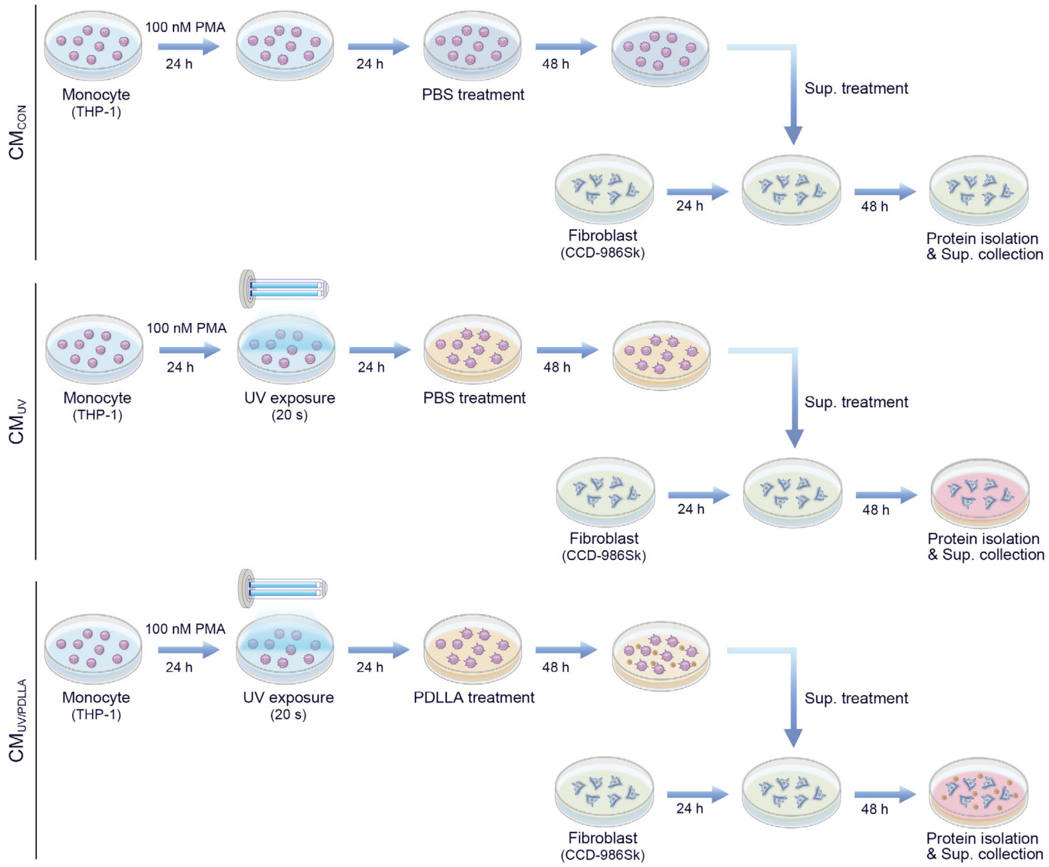


**Figure S4.** Schematic diagram of control fibroblasts ($CM_{CON}$) or UV-irradiated fibroblasts treated with CM collected from macrophages treated with UV and either PBS ($CM_{UV}$) or PDLLA ($CM_{UV/PDLLA}$). CM, conditioned medium; PBS, phosphate-buffered saline; PDLLA, poly-d,l-lactic acid; SD, standard deviation; PMA, phorbol 12-myristate 13-acetate; Sup, supernatant; UV, ultraviolet.

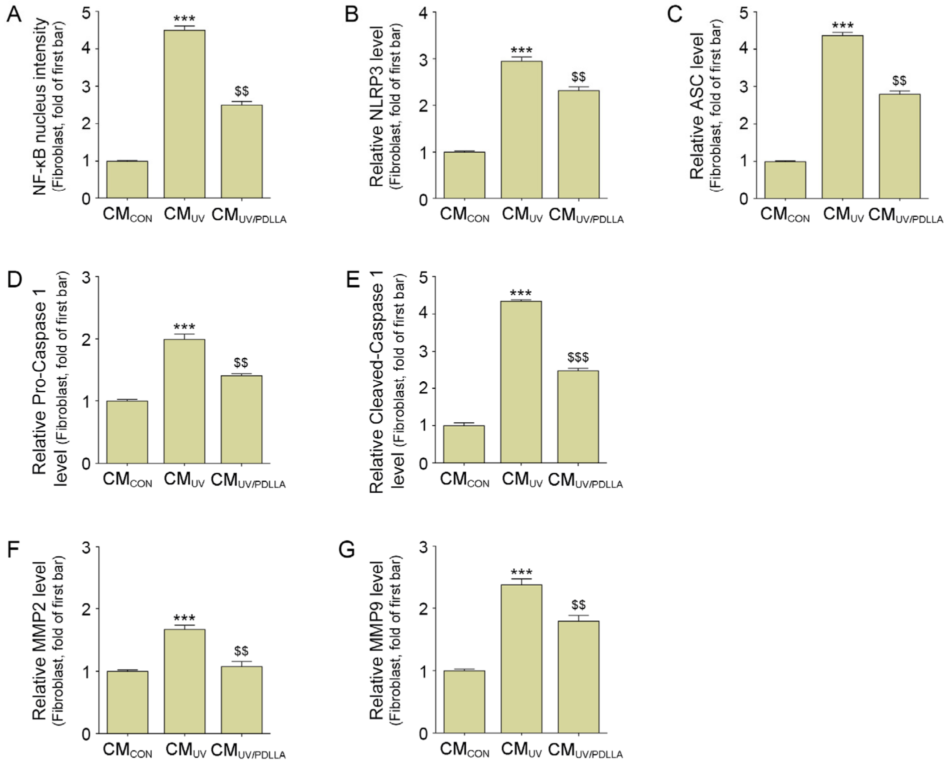


**Figure S5.** Regulation of NLRP3 inflammasome and MMPs expression by PDLLA in fibroblasts treated with $CM_{CON}$, $CM_{UV}$, or $CM_{UV/PDLLA}$. (**A**) Quantitative immunocytochemistry data presented in Figure 2A. (**B–E**) Quantitative western blot data presented in Figure 2B. (**F,G**) Quantitative western blot data presented in Figure 2D. Data are presented as the mean ± SD of three independent experiments. ***, $p < 0.001$, first bar vs. second bar; $$, $p < 0.01$, vs. second bar (Mann–Whitney U test). ASC, apoptosis-associated speck-like protein containing a C-terminal caspase recruitment domain; CM, conditioned medium; CON, control; MMP, matrix metalloproteinase; NF-κB, nuclear factor kappa-light-chain-enhancer of activated B cells; NLRP3, nucleotide-binding domain and leucine-rich repeat pyrin-containing protein 3; PDLLA, poly-d,l-lactic acid; SD, standard deviation; UV, ultraviolet.

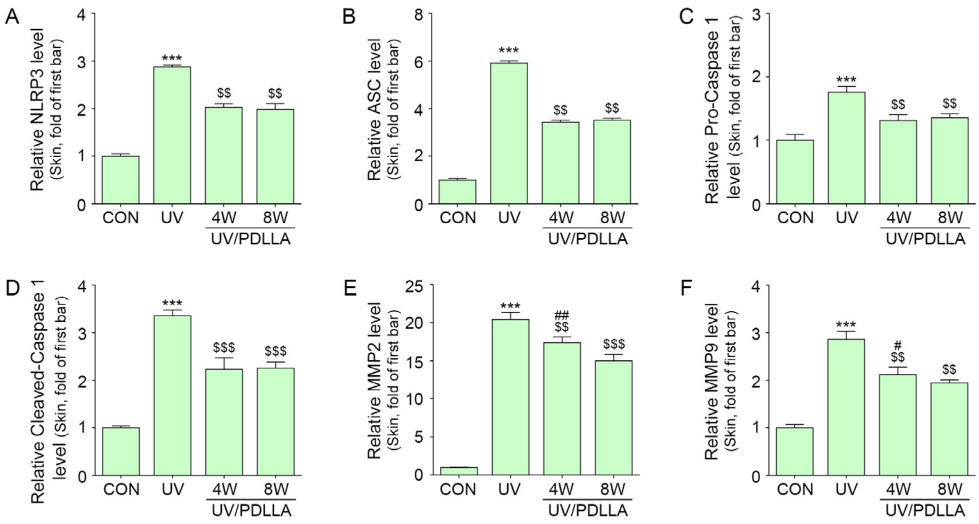


**Figure S6.** Regulation of NLRP3 inflammasome and MMPs expression after PDLLA injection in UVB-irradiated mouse skin. (**A–D**) Quantitative assessment of western blot data presented in Figure 4C. (**E,F**) Quantitative assessment of western blot data presented in Figure 4E. Data presented as the mean ± SD of three independent experiments. ***, $p < 0.001$, first bar vs. second bar; $$, $p < 0.01$, $$$, $p < 0.001$, vs. second bar; #, $p < 0.05$, ##, $p < 0.01$, vs. fourth bar (Mann–Whitney U test). ASC, apoptosis-associated speck-like protein containing a C-terminal caspase recruitment domain; CON, control; MMP, matrix metalloproteinase; MW, molecular weight; NF-κB, nuclear factor kappa-light-chain-enhancer of activated B cells; NLRP3, nucleotide-binding domain and leucine-rich repeat pyrin-containing protein 3; PDLLA, poly-d,l-lactic acid; SD, standard deviation; UV, ultraviolet; W, weeks.

**Table S1.** List of antibodies for enzyme-linked immunosorbent assay (ELISA), western blot (WB) and immunocytochemistry (ICC) / immunohistochemistry (IHC).

| Antibody | Company | Catalog No. | Dilution rate | | |
|---|---|---|---|---|---|
| | | | ELISA | WB | ICC/IHC |
| TNF-α | Santa cruz | Sc-52746 | 1:100 | - | - |
| CD86 | Santa cruz | Sc-19617 | - | 1:500 | |
| CD163 | Santa cruz | Sc-58965 | - | 1:500 | |
| β-actin | Cell signaling | 4967 | - | 1:1000 | - |
| NF-κB | Cell signaling | 8242 | - | - | 1:200 |
| NLRP3 | BosterBio | M00034 | - | 1:500 | - |
| ASC | Santa cruz | Sc-271054 | - | 1:500 | - |
| Caspase 1 | Santa cruz | Sc-392736 | - | 1:500 | - |
| IL-18 | Invitrogen | PA5-79481 | 1:500 | - | - |
| MMP2 | GeneTex | GTX104577 | - | 1:1000 | - |
| MMP9 | Abclonal | A0289 | - | 1:1000 | - |
| Nidogen | Santa cruz | Sc-47773 | 1:500 | - | 1:100 |
| Collagen IV | Invitrogen | PA1-28534 | 1:500 | | 1:100 |
| GP100 | Abcam | Ab137078 | - | 1:1000 | - |
